# A Head-to-Head Comparison of Three Literature Algorithms for Physical Activity Endpoints from a Wrist Accelerometer in Free-Living Conditions

**DOI:** 10.3390/s26165194

**Published:** 2026-08-17

**Authors:** Nunzio Camerlingo, Lily Koffman, Lukas Adamowicz, Evgenia Moustridi, Fikret Isik Karahanoglu

**Affiliations:** 1 Pfizer Research and Development, Cambridge, MA 02139, USA; 2 Department of Biostatistics, Johns Hopkins Bloomberg School of Public Health, Baltimore, MD 21205, USA

**Keywords:** digital health, accelerometers, physical activity endpoints, SciKit digital health

## Abstract

**Highlights:**

**What are the main findings?**

- A comparative evaluation of three established algorithms for deriving physical activity endpoints from wrist-worn accelerometer data demonstrates meaningful variability in performance across methodologies and activity intensities.
- A threshold-based algorithm (Adamowicz et al.) demonstrated the most balanced and consistent performance across activity intensities.

**What are the implications of the main findings?**

- Simpler and interpretable approaches can match or exceed more complex machine-learning models in terms of generalizability and bias, supporting their use in regulated and clinical settings.
- Methodological transparency and rigorous validation against gold standard measures (such as calorimetry) should be adopted prior to selecting a fit-for-purpose algorithm and deploying the resulting physical activity endpoints in clinical trials.

**Abstract:**

Wrist accelerometers are widely used in clinical trials for continuous assessment of participants’ day-to-day physical activity and function, through derivation of metrics such as time spent in sedentary, non-sedentary, light, and moderate-to-vigorous physical activity (MVPA). Multiple algorithms have been proposed to derive these physical activity endpoints; however, the agreement across the endpoints derived from these algorithms has not been thoroughly explored. In this work, we leverage a publicly available dataset, CAPTURE-24, including wrist accelerometer data and experts’ annotations of various physical activities for 151 healthy adults, monitored at-home for one day, to compare three literature algorithms: Algorithm #1, SciKit Digital Health (SKDH), from Adamowicz et al., Algorithm #2 from Staudenmayer et al., and Algorithm #3 from Montoye et al. Algorithms’ outputs were assessed against experts’ annotations via paired *t*-tests, Pearson’s correlation (R), Bland–Altman analysis, accuracy, sensitivity, specificity, and F1-score. Algorithm #1 and Algorithm #2 showed stronger correlation and lower bias with experts’ annotations for time in sedentary (Algorithm #1: R = 0.91), and MVPA (Algorithm #1: R = 0.63), compared to Algorithm #3. In addition, Algorithm #1 and Algorithm #3 outperformed Algorithm #2 for time in light activity. Future work using longer monitoring periods and reference calorimetry devices should be performed to confirm the reproducibility of these findings.

## 1. Introduction

A physically active lifestyle is strongly associated with a decreased risk of a wide range of chronic diseases and adverse health conditions, including cardiovascular diseases, hypertension, obesity, type 2 diabetes, cancer, and depression [1,2,3,4]. In contrast, physical inactivity is unequivocally recognized as a leading risk factor for all-cause mortality, with estimates exceeding 5 million deaths per year [5], and a major contributor to the global burden of disease, especially for chronic diseases, with increasing impact worldwide [6]. In response to this evidence, the World Health Organization (WHO) recommends that adults perform at least 150 min of moderate or 75 min of vigorous physical activity per week, or a combination of both, to achieve meaningful health benefits [7,8]. Consequently, in clinical practice and preventive medicine, physical activity is considered a cornerstone of lifestyle assessment and intervention, alongside other key behavioral factors, such as smoking, alcohol consumption, and diet [9].

Traditionally, physical activity and function in clinical trials have been assessed using self-reported questionnaires, activity diaries, and in-lab assessments, such as the Timed-Up-And-Go (TUG), six-minute walk test (6MWT), and the Short Physical Performance Battery (SPPB) [10]. While these methods are widespread and generally convenient for sites to administer, they are prone to recall bias (or white coat effect) and capture episodic evaluation that does not reflect day-to-day life. This can lead to substantial misinterpretation of the outcomes and limited sensitivity to change [11]. These limitations have become increasingly salient as clinical research shifts toward more sensitive, patient-centered, and real-world endpoints. Recent advances in digital health technologies (DHTs), including wearable accelerometers, have enabled continuous and objective assessment of physical activity, providing information on movement patterns, intensity, duration, and variability over extended periods of time, in both controlled and free-living environments [12,13].

Wrist-worn accelerometers record tri-axial acceleration signals, capturing the direction and magnitude of movement, which are subsequently aggregated over fixed duration time window (e.g., 15 s, 1 min) into summary metrics such as activity counts [14], vector magnitude [15], or Euclidean norm minus one (ENMO) [16]. These metrics are widely used proxies for physical activity intensity. Based on these derived measures, time spent in different activity intensity categories can be quantified. Periods characterized by very low intensity values are classified as sedentary, typically reflecting activities with minimal energy expenditure, such as sitting or lying down [17]. Activities such as washing dishes, watering plants, or writing generate higher acceleration signals, and are generally categorized as light physical activity. Finally, moderate-to-vigorous physical activity (MVPA) typically corresponds to activities associated with substantially higher energy expenditure, including brisk walking, stair climbing, or running.

Beyond improving the characterization of activities of daily living and their relationship with health outcomes, the integration of accelerometer-derived physical activity endpoints into interventional clinical trials offers several critical advantages, including improved sensitivity to treatment, higher statistical power, reduced sample size, and enhanced participant accessibility and ecological validity via decentralized or hybrid trial designs [18,19,20]. Reflecting this paradigm shift, regulatory agencies have begun to formally recognize accelerometer-derived endpoints [21]. Notably, in 2019, the 95th percentile of stride velocity received formal qualification from the European Medicine Agency (EMA) to be used as a primary endpoint for ambulatory patients with Duchenne muscular dystrophy. Later, in 2022, the U.S. Food and Drug Administration (FDA) endorsed MVPA as the primary endpoint in the pivotal Phase III REBUILD study investigating the treatment of pulmonary fibrosis, where its use led to a substantial reduction in required sample size [19]. More recently, time spent in non-sedentary activity demonstrated a statistically significant treatment effect of a novel therapeutic agent in cancer cachexia [22].

The growing regulatory acceptance and scientific interest in physical activity digital endpoints have intensified the need for robust, transparent, and validated algorithms to derive clinically meaningful measures from raw accelerometer data. Numerous algorithms have been proposed in the literature for this purpose, varying in signal processing techniques, feature extraction, intensity thresholds, and device-specific assumptions. The development and validation of these algorithms require reference measures of energy expenditure or activity intensity, such as calorimetry devices [23], or standardized resources like the Compendium of Physical Activities [17]. In validation studies, participants instrumented with wearable accelerometers perform a series of predefined tasks spanning a broad range of intensities. These tasks are subsequently labeled as sedentary, light, or MVPA, using established thresholds [24], which may vary according to individual characteristics such as age, physiological capacity, or the presence of specific diseases. Finally, the algorithm-derived activity categories are compared against the reference labels to assess classification accuracy, agreement, and generalizability [25,26,27,28].

The validation studies are typically designed for testing a newly developed algorithm or wearable accelerometer; therefore, multiple algorithms might not be assessed simultaneously. In order to assess the consistency of physical activity endpoints derived by multiple algorithms, we used the publicly available CAPTURE-24 dataset, consisting of wrist accelerometer data from individuals performing different activities and expert-annotated activity categories, to compare physical activity endpoints derived from three literature algorithms: Adamowicz et al., [29], Staudenmayer et al. [30], and Montoye et al. [31]. These selected algorithms span different methodologies to derive physical activity endpoints. Adamowicz et al. is a simple and flexible threshold-based approach, developed in-house and publicly available. Staudenmayer et al. and Montoye et al. both leverage machine learning approaches: the former is a multi-stage interpretable machine learning algorithm, using time and frequency domain features, and inspiring the logic of certain commercially available devices (such as Ametris watch, Pensacola, FL, USA). The latter is an advanced machine learning algorithm using only time domain features to estimate activity intensity directly, made publicly available.

## 2. Materials and Methods

### 2.1. Dataset: CAPTURE-24

The open-source CAPTURE-24 dataset [32] is a large resource designed to support the development and validation of physical activity algorithms derived from wearable accelerometers in free-living conditions. The dataset includes 151 healthy adult participants (99 females, aged 18 years and older) recruited from the general population, monitored continuously for approximately 24 h during their normal daily routines. Participants wore a wrist-worn accelerometer (AX3, Axivity Ltd., Newcastle upon Tyne, UK), which recorded tri-axial acceleration at 100 Hz with a dynamic range of ±8 g, providing high-resolution raw movement data suitable for activity intensity and human activity recognition analyses. To obtain ground-truth activity annotations, participants simultaneously wore a wearable camera (Vicon Autographer, OMG Life, Oxfordshire, UK) around the neck, during waking hours only, and completed sleep diaries to identify periods of sleep. The wearable camera automatically captured first-person images approximately every 20–40 s, yielding a rich visual record of daily activities. These images were subsequently reviewed and manually annotated by pre-trained human annotators, following a standardized protocol. Activities were labeled at a fine-grained level as specific tasks (e.g., sitting, cooking, walking, cycling), resulting in a total of 2562 h of annotated data. All annotated activities were mapped to standardized activity codes from the Compendium of Physical Activities [17], enabling harmonization across participants and consistency with established metabolic frameworks. Each activity code is associated with an estimated Metabolic Equivalent of Task (MET) value, providing a quantitative measure of energy expenditure. MET values were subsequently collapsed into the following activity categories: sedentary activity, light activity, and MVPA. On mean ± SD across participants, the dataset included 10.49 ± 2.87 h/day of awake time, annotated as follows: 6.60 ± 2.56 h/day sedentary activity, 3.09 ± 1.82 h/day light activity, and 0.80 ± 0.89 h/day MVPA. This distribution reflects realistic free-living behavior patterns and highlights the pronounced class imbalance of real-world physical activity data, with sedentary and light activities dominating the daily time.

### 2.2. Algorithm #1: Adamowicz et al., 2022

Numerous algorithms have been proposed in the literature to derive physical activity categories with a threshold-based approach. Some of these algorithms differ on the metrics used to aggregate raw acceleration data into measures of activity intensity, such as Euclidean Norm Minus One (ENMO) [29,33], activity counts [34,35] (common in Actigraph devices) and Sum of Vector Magnitude [27,36] (common in GENEActiv devices). Other threshold-based algorithms first perform a calibration from tri-axial accelerometer data to MET, and then use thresholds on these values [37]. Furthermore, other approaches look at the variability of the raw acceleration, quantified for example via mean amplitude deviation [38] or activity index [39]. Finally, when gait is a measurement of interest, some algorithms implement thresholds on the number of steps [40]. In this work, we selected the threshold-based algorithm from SciKit Digital Health (SKDH), an open-source, device-agnostic, modular, and extensible framework developed to address the growing need for transparent, reproducible, and interoperable analytics pipelines in digital health research [29]. The framework supports the standardized ingestion, processing, and analysis of wearable inertial sensor data for the derivation of digital endpoints across multiple domains of measurement, including physical activity, gait, sit-to-stand, and sleep, and across different body positions [41].

In the physical activity module, raw tri-axial accelerometer data collected from wrist-worn devices are first calibrated, processed, and aggregated over fixed-duration epochs (e.g., 15 s duration). Physical activity intensity is then quantified using ENMO (mg), which provides an orientation-independent estimate of the movement intensity. ENMO values are subsequently classified into mutually exclusive physical activity intensity categories, using a threshold-based approach.

In this work, physical activity categories were derived using thresholds proposed by Migueles et al. [42]. Specifically, epochs with ENMO < 50 mg are classified as sedentary activity, 50 mg < ENMO < 110 mg as light activity, 110 mg < ENMO < 440 mg as moderate activity, and ENMO ≥ 440 mg as vigorous activity.

The performance and validity of SKDH-derived physical activity measures have been evaluated by Lin et al. [43], who assessed SKDH outputs against two widely used accelerometry analysis tools: GGIR and GENEActiv Macros. Results showed that across key physical activity endpoints—including time in sedentary activity, light activity, and MVPA—SKDH demonstrated high pairwise agreement, strong correlation, and minimal systematic bias relative to comparator pipelines, supporting the methodological equivalence of physical activity endpoints across well-established analysis tools.

### 2.3. Algorithm #2: Staudenmayer et al., 2015

Staudenmayer et al. [30] developed a set of supervised statistical learning models to derive physical activity endpoints from wrist-worn accelerometer data, with the goal of improving activity classification and energy expenditure estimation under free-living conditions. In their framework, raw tri-axial accelerometer data are segmented into non-overlapping 15 s windows, within which a comprehensive set of time-domain and frequency-domain features is extracted, capturing signal magnitude, variability, and spectral characteristics associated with different movement intensities.

These features are then fed to two distinct decision-tree-based classifiers that were developed: the first model assigns each time window to the light, moderate, or vigorous physical activity category, while the second model provides a binary classification of sedentary versus non-sedentary behavior. This dual-model approach allows each 15 s window to be simultaneously characterized by both decision trees, therefore potentially allowing one epoch to be classified both as sedentary and as light/moderate/vigorous. Finally, the classified epochs are summed to obtain the daily aggregated endpoints.

Model training and validation were conducted using data from 20 healthy adult participants who completed a structured protocol consisting of a broad range of activities spanning sedentary behaviors, ambulatory tasks, and higher-intensity exercises, while wearing a wrist accelerometer and indirect calorimetry as reference device. The sedentary vs. non-sedentary classifier demonstrated particularly strong performance, achieving classification accuracy exceeding 95% for sedentary detection [30], while the multi-class intensity classification showed good agreement across light, moderate, and vigorous activity categories.

### 2.4. Algorithm #3: Montoye et al., 2018

Montoye et al. [31] proposed and systematically evaluated a series of machine-learning models for classifying physical activity intensity categories from wrist-worn accelerometer data. In their framework, raw tri-axial acceleration signals were segmented into 30 s non-overlapping windows, from which multiple sets of representative features were extracted, including time-domain features (e.g., signal magnitude, variability, and distributional statistics) and frequency-domain features (reflecting different aspects of movement intensity and periodicity).

The study benchmarked the performance of several commonly used classification algorithms, including random forest (RF), single decision tree, artificial neural network, and support vector machine (SVM), across different feature sets. Two independent datasets were used for model development and evaluation: the first dataset (*n* = 39) was used for training and internal validation, and the second dataset (*n* = 24) was used exclusively for external testing, enabling a rigorous assessment of out-of-sample generalizability.

Across algorithms and feature sets, the RF paired with time-domain features alone demonstrated superior classification performance, without the need for more computationally intensive frequency-domain inputs. In the external validation, the RF achieved the highest overall accuracy in the classification of epochs as sedentary, light, moderate, or vigorous, ranging from 77.3% to 78.5%, depending on the specific activity grouping [31]. Notably, Montoye et al. also compared the machine learning classifiers against the widely used GGIR package, showing that the RF outperformed GGIR in terms of classification accuracy.

### 2.5. Statistical Analysis

Algorithm #1 and Algorithm #2 are implemented in Python (version 3.11, while Algorithm #3 is made available by the authors in R (version 4.5.2).

For Algorithm #2, time and frequency domain features are computed, including the standard deviation (SD) of vector magnitude (defined as the Euclidean norm of the raw accelerometry data), and the percent power for the Fourier transform of the vector magnitude within 0.6–2.5 Hz (see [30] for a comprehensive list). For Algorithm #3, the following features were computed for each of the three directions: mean, median, SD, minimum, maximum, 10th, 25th, 75th, and 90th percentile.

Raw accelerometer data from the CAPTURE-24 dataset are aggregated into 15 s windows to run Algorithm #1 and Algorithm #2, and also into 30 s windows to run Algorithm #3. To ensure compatibility between the different window sizes, the 30 s prediction obtained from Algorithm #3 is split into two 15 s windows, assuming constant physical activity.

The performance of the algorithms is evaluated at two temporal resolutions: daily aggregates and 15 s epochs. At the daily level, time spent in each of the following physical activity categories (h/day) is computed for each participant and algorithm: sedentary, light, moderate, vigorous, MVPA (defined as the sum of moderate and vigorous), and non-sedentary (defined as the sum of light, moderate, and vigorous for Algorithm #1 and Algorithm #3, and given as a byproduct of the sedentary vs. non-sedentary classification for Algorithm #2). The comparison between algorithm-derived estimates and experts’ annotations is conducted via paired *t*-tests or Wilcoxon signed-rank test (based on the normality of the paired differences, assessed via Shapiro–Wilk test at a 5% significance level), scatter plots with Pearson’s correlation (R), and Bland–Altman analysis reporting bias (Δ) with 95% Limits of Agreement (LoA). *p*-values were adjusted for multiple comparisons using the False Discovery Rate (FDR) method. Pearson’s R is evaluated using the guidelines from Chan et al. [44]: R ≥ 0.8: strong correlation, 0.8 > R ≥ 0.6: moderate correlation, 0.6 > R ≥ 0.3: fair correlation, R < 0.3: poor correlation. At the 15 s epoch level, a binary classification approach is used to evaluate the algorithms’ performances in classifying sedentary vs. non-sedentary, light vs. non-light, and MVPA vs. non-MVPA with respect to the experts’ annotations. For each of these classification problems, the number of true positives (TP), true negatives (TN), false positives (FP), and false negatives (FN) is computed per participant, and the mean, together with the 95% confidence interval (CI) is reported in confusion matrices, assuming a prediction equal to 1 for sedentary/light/MVPA and equal to 0 for non-sedentary/non-light/non-MVPA. The following summary metrics are computed per participant: accuracy, sensitivity, specificity, precision, and F1-score. The area under the receiver operating characteristic curve (AUROC) is also estimated as the area of the triangle defined by the points (1-specificity, sensitivity), (0, 0), and (1, 1), using the following formula:

$$AUROC\approx \frac{\left(1-specificity\right)\times sensitivity}{2}+\frac{\left(1+sensitivity\right)\times specificity}{2}.$$

This measure represents the average of sensitivity and specificity, i.e., the weighted accuracy, which gives equal weight to the positive and negative class and therefore should be preferred to accuracy in those classification problems affected by class imbalance.

To account for inter-individual differences in the number of valid epochs, all performance metrics are first computed within each participant and then summarized across participants to obtain population-level estimates.

## 3. Results

### 3.1. Comparison over Daily Aggregates

The daily time spent in sedentary, light, MVPA, and non-sedentary activity is reported in Table 1, for the experts’ annotations and the algorithms under evaluation. For each activity category, the *p*-values of the paired *t*-test between the experts’ annotations and the algorithm’s predictions are also reported.

Predictions from all the algorithms under analysis are significantly different from experts’ annotations for all the physical activity categories. Algorithm #1 notably shows similar ranking of average daily time spent in the different activity ranges: longer time is spent in sedentary activity, followed by light and MVPA. For Algorithm #2, the average daily time in light activity is heavily overestimated compared to the experts’ annotations (8.90 [7.32, 10.5] h/day vs. 2.74 [1.68, 4.36] h/day, as median [IQR]). Finally, Algorithm #3 shows higher average daily time in MVPA compared to experts’ annotations (0.979 [0.401, 2.62] h/day vs. 0.504 [0.174, 1.12] h/day).

**Figure 1:**
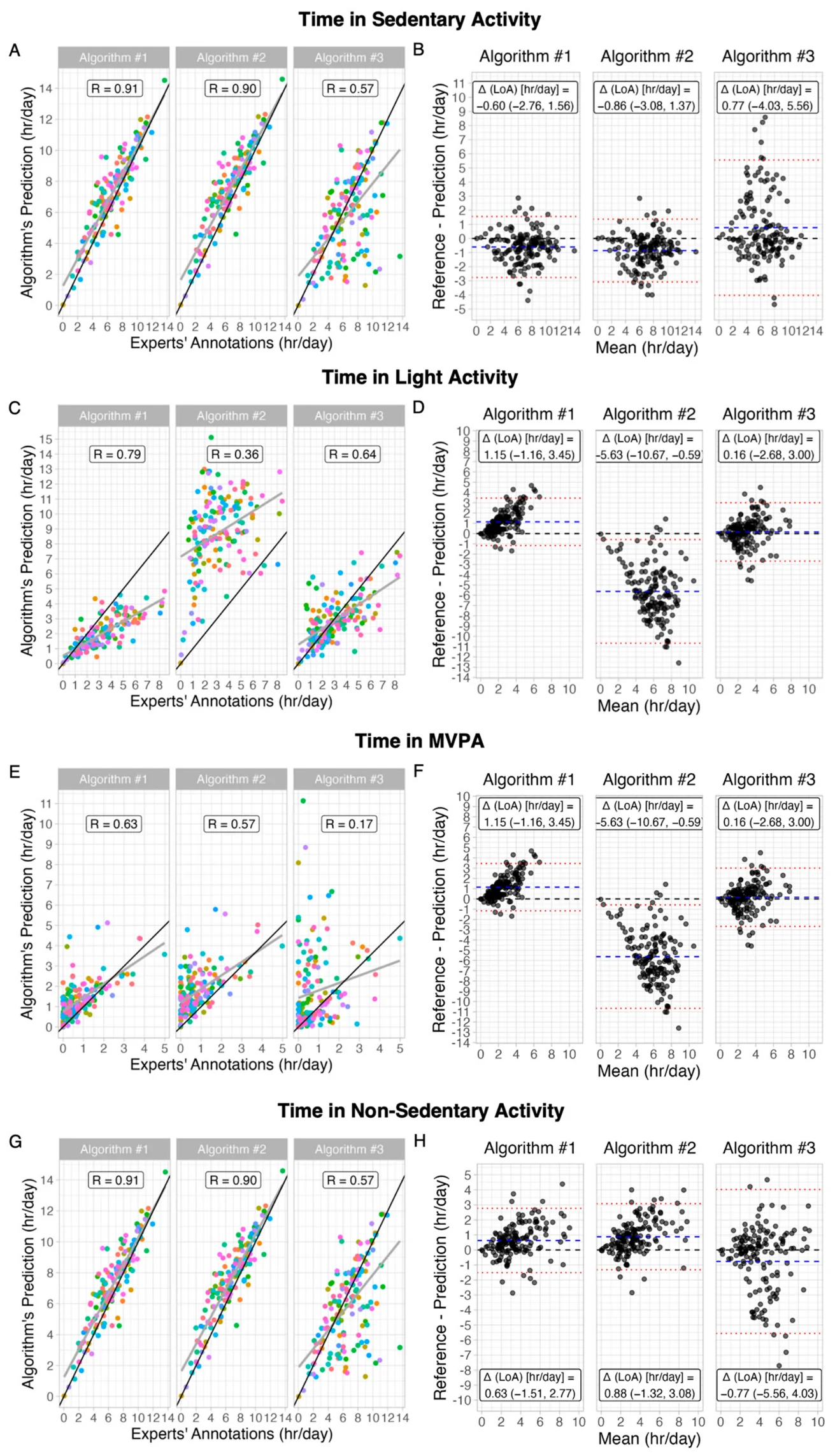
Scatter plots with Pearson’s R (panels (**A**,**C**,**E**,**G**)) and Bland–Altman plots with bias (Δ) and 95% LoA (panels (**B**,**D**,**F**,**H**)), between the algorithms under analysis versus the experts’ annotations, for time in sedentary (panels (**A**,**B**)), light (panels (**C**,**D**)), MVPA (panels (**E**,**F**)), and non-sedentary (panels (**G**,**H**)) activity. In the scatter plots, participants are color-coded, regression line and R = 1 are reported in gray and black, respectively. In the Bland–Altman plots, the bias, Δ = 0, and 95% LoA are reported as blue, black, and red dashed lines, respectively. Algorithm #1: Adamowicz et al.; Algorithm #2: Staudenmayer et al., Algorithm #3: Montoye et al. LoA: Limits of Agreement; MVPA: moderate-to-vigorous physical activity.

Scatter plots with Pearson’s R and Bland–Altman plots with bias and LoA between the algorithms’ predictions and the experts’ annotations are reported in Figure 1 for the daily time spent in sedentary, light, MVPA, and non-sedentary activity.

For time in sedentary activity, Algorithm #1 and Algorithm #2 show similar daily predictions compared to experts’ annotations, with a strong correlation (R = 0.91 and R = 0.90, respectively) and homogeneous bias characterized by an overestimation of less than 1 h/day (Δ = −0.60 h/day, Δ = −0.86 h/day, respectively). Algorithm #3 shows a lower correlation (R = 0.57) and a positive bias with larger LoA (Δ = 0.77 h/day, LoA: (−4.03, 5.56) h/day).

For time in light activity, Algorithm #1 and Algorithm #3 exhibit a moderate correlation (R = 0.79 and R = 0.64, respectively), and small underestimation (Δ = 1.15 h/day, Δ = 0.16 h/day, respectively). Bias is homogeneous for Algorithm #3, while it shows a positive trend for Algorithm #1. Algorithm #2 exhibits a lower correlation (R = 0.36) and a remarkably non-homogeneous bias (Δ = −5.63 h/day), with LoA lower than the 0-threshold and high overestimation of larger values of time in light activity (for example, mean values of time in light of ~7 h/day are associated with a mean bias of ~−10 h/day).

For time in MVPA, Algorithm #1 and Algorithm #2 present similar correlations: moderate for Algorithm #1 (R = 0.63) and fair for Algorithm #2 (R = 0.57). Bland–Altman analyses lead to similar outcomes, with homogeneous, negative bias, larger for Algorithm #2 (Δ = −0.95 h/day) than Algorithm #1 (Δ = −0.52 h/day). Algorithm #3 presents a poor correlation (R = 0.17) and negative non-homogeneous bias with larger LoA (Δ = −0.93 h/day, LoA: (−4.79, 2.94) h/day).

Lastly, for time in non-sedentary activity, Algorithm #1 and Algorithm #2 show similar daily predictions compared to experts’ annotations, with a strong correlation (R = 0.86 for both algorithms) and homogeneous, positive bias, larger for Algorithm #2 (Δ = −0.88 h/day) than Algorithm #1 (Δ = −0.63 h/day). Algorithm #3 shows a fair correlation (R = 0.46) and negative, homogeneous bias, with larger LoA (Δ = −0.77 h/day, LoA: (−5.56, 4.03) h/day).

### 3.2. Comparison over 15 s Epochs

**Figure 2:**
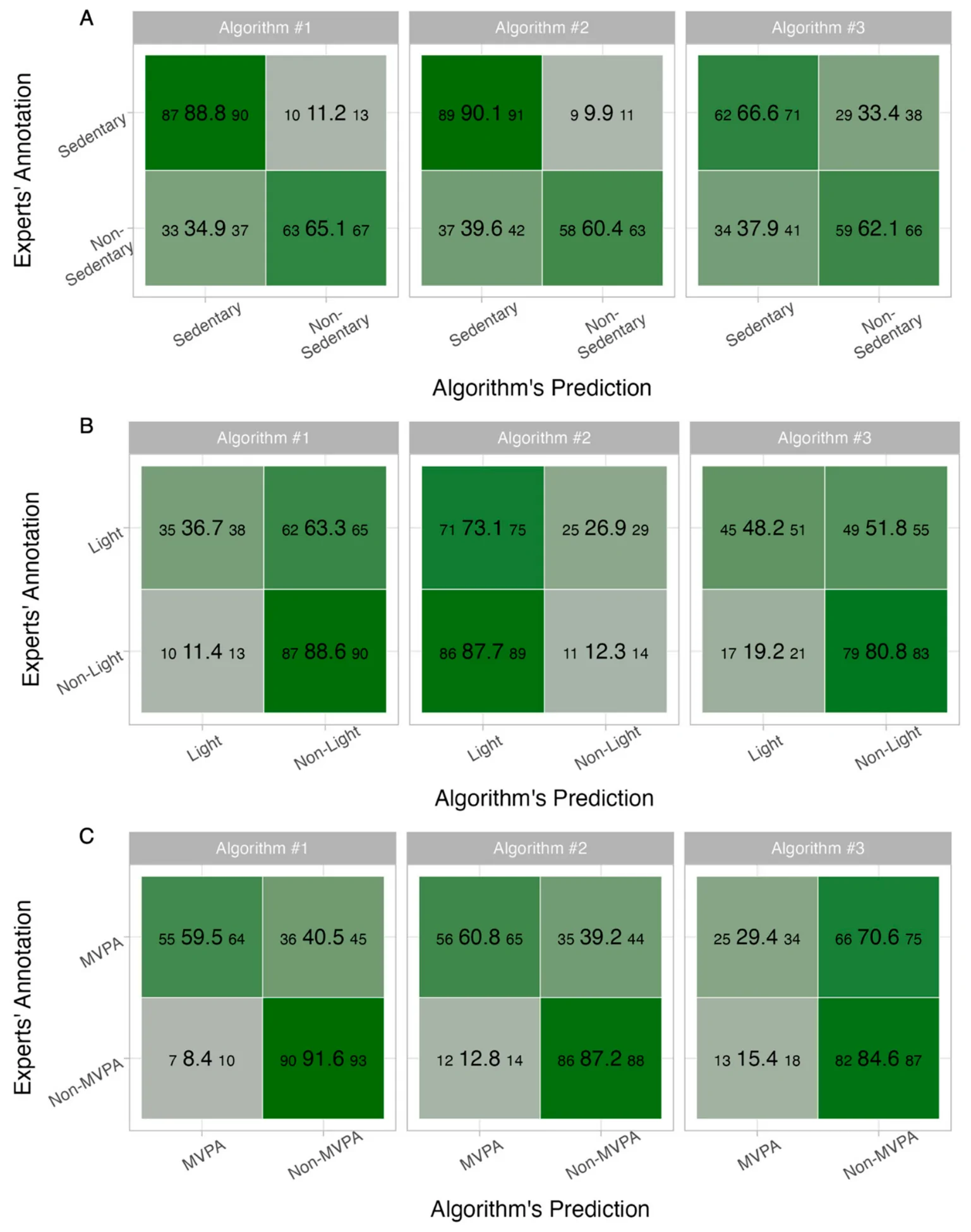
Confusion matrices between algorithms’ predictions and experts’ annotations for the binary classification of sedentary vs. non-Sedentary (panel (**A**)), light vs. non-light (panel (**B**)), MVPA vs. non-MVPA (panel (**C**)). Tiles report percent of TP, TN, FP, and FN as lower bound of 95% CI, mean, and upper bound of 95% CI across participants, obtained from the 15 s epochs. Algorithm #1: Adamowicz et al.; Algorithm #2: Staudenmayer et al.; Algorithm #3: Montoye et al.; MVPA: moderate-to-vigorous physical activity; CI: confidence interval; TP: true positives; TN: true negatives; FP: false positives; FN: false negatives.

The confusion matrices between the experts’ annotations and the algorithms’ predictions are reported in Figure 2 for the three binary classification problems: sedentary vs. non-sedentary, light vs. non-light, and MVPA vs. non-MVPA. The percent of TP, TN, FP, and FN across participants is reported in each tile as mean and (95% CI). Furthermore, summary classification metrics are reported as mean ± SD across participants in Table 2.

For the sedentary vs. non-sedentary classification, Algorithm #1 shows high sensitivity, with 88.8% (87, 90)% of TP, and high specificity, with 65.1% (63, 67)% of TN. The percent of TP is slightly higher for Algorithm #2, equal to 90.1% (89–91)%, while the percent of TN is slightly lower, at 60.4% (58–63)%. Algorithm #3 has a similar percent of TN, but lower percent of TP, with 33.4% (29–38)% of Sedentary epochs classified as non-sedentary. Algorithm #1 and Algorithm #2 have similar accuracy (0.809 ± 0.080 and 0.798 ± 0.077, respectively, with respect to a percent of positive class of 63.0% ± 16.8%, as mean ± SD across participants) and F1-score (0.838 ± 0.096 and 0.833 ± 0.093, respectively), while AUROC is slightly higher for Algorithm #1 (0.770 ± 0.077) compared to Algorithm #2 (0.752 ± 0.073).

For the light vs. non-light classification, Algorithm #1 and Algorithm #3 exhibit high specificity, with percent of TN equal to 88.6% (87–90)% and 80.8% (79–83)%, respectively, but moderate sensitivity, with average percent of TP lower than 50% for both. Algorithm #2 exhibits better sensitivity (percent TP equal to 73.1% (71–75)%), but also a remarkably high percent of FN, with 87.7% (86–89)% of non-light epochs classified as light. Algorithm #1 and Algorithm #3 are similar in terms of F1-score (0.438 ± 0.107 and 0.478 ± 0.208, respectively) and weighted accuracy (0.627 ± 0.053 and 0.645 ± 0.128, respectively), while accuracy is affected by the class imbalance (with the percent of positive class of 29.3% ± 14.8%).

For the MVPA vs. non-MVPA classification, all algorithms show high percentage of TN, with the highest observed for Algorithm #1, equal to 91.6% (90–93)%. While Algorithm #1 and Algorithm #2 show similar percentage of TP, equal to 59.5% (55–64)% and 60.8% (56–65)%, respectively, for Algorithm #3, 70.6% (66, 75)% of the epochs annotated as MVPA are mistakenly classified as non-MVPA, resulting in the highest percentage of FN across the algorithms and endpoints. Algorithm #1 shows a weighted accuracy slightly higher than that of Algorithm #2 (0.755 ± 0.148 vs. 0.740 ± 0.146), while accuracy is affected heavily by the class imbalance (with the percent of positive class of 7.70% ± 8.00%). Algorithm #1 shows an F1-score of 0.453 ± 0.256 that, despite being low in magnitude, is the highest across the algorithms under comparison. The discrepancy in magnitude between F1-score and other metrics for the light vs. non-light and MVPA vs. non-MVPA classification problems reflects the inherent class imbalance, with the light and MVPA classes less prevalent than non-light and non-MVPA ones, respectively. The lower F1-score suggests that most classification errors occurred in identifying the positive class rather than in distinguishing active from inactive behavior overall. Specifically, for Algorithm #2, the low F1-scores in the light vs. non-light and the MVPA vs. non-MVPA classifications are driven by a lower precision, suggesting over-classification of the target class, as also visible in Table 1. For Algorithm #3, the low F1-score for the MVPA vs. non-MVPA classification, in addition to the low sensitivity and low precision, suggests that it over-classifies MVPA epochs (as confirmed by Table 1), and also misses the true MVPA labels.

## 4. Discussion

Multiple algorithms have been proposed in the literature to derive physical activity endpoints from wrist-worn accelerometer data. These approaches differ along several methodological dimensions, including the signal pre-processing pipeline (e.g., filtering, calibration, and temporal aggregation), the classification strategy (e.g., threshold-based versus data-driven machine learning methods), the feature representation (e.g., time-domain or frequency-domain) and the validation framework (e.g., using a calorimetry device or the Compendium of Physical Activities). In this study, we evaluated the performance of three previously published algorithms: Algorithm #1 from Adamowicz et al. [29], Algorithm #2 from Staudenmayer et al. [30], and Algorithm #3 from Montoye et al. [31], each originally assessed using distinct validation frameworks and datasets. While a fully controlled comparison would ideally require a dedicated experimental study, we leveraged the publicly available CAPTURE-24 dataset, which provides free-living, expert-annotated data from a wrist-worn accelerometer. The evaluation revealed that models differ in terms of what component of daily physical activity can be more accurately represented, demonstrating that not all models are equally equipped to handle the complexity of daily human movement. At the daily aggregation level, Algorithm #1 demonstrated the most consistent and strong correlations with the annotated reference across sedentary, light, and MVPA ranges, with minimal bias. Algorithm #2 performed similarly well for sedentary activity, but lacked precision in differentiating intermediate activities, substantially overestimating time in light activity (with a bias of 5.63 h/day). This is driven by the intrinsic use of two separate decision tree classifiers, one for sedentary vs. non-sedentary and one for light vs. MVPA, which results in the assignment of each time epoch to two potentially different activity ranges. As a consequence, Algorithm #2 has very low specificity for the light vs. non-light classification, because the decision tree that provides this prediction does not account for the presence of a sedentary class; therefore, most of the sedentary epochs labeled as non-light are likely misclassified as light activity. Furthermore, Algorithm #3 shows a small average bias, in line with that of Algorithm #1 and Algorithm #2, but higher variance and LoA, suggesting that the algorithm performance might not be consistent across participants, especially in some participants spending longer sedentary time. Finally, Algorithm #3 yielded reasonable performance for light activity (exhibiting the lowest average bias), but displayed limited utility for higher intensity activities. This aligns with the findings previously obtained by Montoye and colleagues [31], who attributed a lower performance in the MVPA vs. non-MVPA classification to a limited representation of MVPA across age groups in the datasets used for training the models. For example, the authors claimed that the motion observed in older participants performing vigorous activities, such as jogging, was similar to that of younger participants performing lighter activities, such as brisk walking, which might drive the lower sensitivity and higher bias observed in our work. A more granular evaluation at the 15 s epoch level further highlighted differences in binary classification performance. Both Algorithm #1 and Algorithm #2 achieved superior performance relative to Algorithm #3 for the sedentary vs. non-sedentary and the MVPA vs. non-MVPA classifications. In addition, Algorithm #1 and Algorithm #3 achieved superior performance relative to Algorithm #2 for the light vs. non-light classification.

In summary, this comprehensive comparison demonstrates that while no single algorithm achieves perfect accuracy across all intensities, the framework presented in Algorithm #1 provides a highly balanced and reliable method for quantifying daily physical activity patterns. These findings reinforce and expand upon current knowledge regarding accelerometer-based physical activity classification, highlighting that threshold-based algorithms, like Algorithm #1, can achieve competitive (and in some categories superior) reliability without the computational complexity of black-box models, such as the foundational machine learning benchmarks established by Willetts et al. [45] and Montoye et al. [31]. The performance of Algorithm #1 validates the continued relevance of transparent methodologies, as already proposed by Hildebrand et al. [33] and van Hees et al. [16], highlighting the ability of these algorithms to effectively navigate the challenges of free-living data, supporting the integration of clinically relevant digital health endpoints into large-scale clinical trials.

To contextualize the relevance of these findings, several limitations have to be acknowledged. First, our evaluation relies on a short (24 h) monitoring period per participant, which does not offer the possibility to assess the day-to-day reproducibility and long-term stability of these findings, jeopardizing the generalizability of the results to assess the overall physical activity patterns captured by DHTs, where the measurements were captured over multiple days of continuous monitoring. Furthermore, the reference activity for this dataset was established via human annotation of wearable camera images. While potentially detailed, the original CAPTURE-24 manuscript lacks relevant details on how annotation was performed. Moreover, this method is fundamentally different from a direct measurement of energy expenditure, such as indirect calorimetry, which remains the recognized gold standard for metabolic validation. Consequently, the observed discrepancies between algorithms’ outputs and annotations should be interpreted in the context of uncertainty in the reference labels. In addition, we encountered a pervasive challenge regarding class imbalance: free-living data naturally features a heavy predominance of sedentary and light activities, but the limited occurrence of MVPA epochs notably leads to limitations in sensitivity across all three algorithms. Finally, the results observed for Algorithm #3 might be affected by the assumption that the activity classification provided by the model every 30 s is constant until the next prediction, which might not be true in free-living conditions.

From a regulatory perspective, the present comparison is directly relevant to the evolving FDA framework for wearable technologies and novel digital endpoints [46]. The FDA has supported the deployment of digital health technologies in clinical investigations, but only when they are shown to be fit for purpose for the intended context of use, including evidence across clinical validation [47] and technical validation of the full processing pipeline [48], as well as the quantification of the measurement error (such as bias, variability, and potential drifts), as these factors directly affect endpoint reliability and sensitivity to treatment effects [48,49]. Within this framework, the performance observed across algorithms should be interpreted as part of the analytical validation evidence needed before such endpoints can be deployed as primary or key secondary outcomes. More broadly, our findings support the FDA’s emphasis on transparent, well-characterized algorithms and explicit quantification of uncertainty as prerequisites for the successful use of wearable-derived digital endpoints in clinical drug development.

Ultimately, these findings can be leveraged to inform the design, sample size, and statistical analysis of future research studies, prioritizing the validation of physical activity algorithms over extended monitoring durations, within diverse clinical populations, and using a calorimetry device as the gold standard reference device. Doing so will be critical to establishing the broader generalizability of these powerful digital health tools and ensuring their effective application in personalized medicine and public health monitoring.

## Figures and Tables

**Table 1:** Daily time spent in different physical activity categories (h/day) across classification strategies., reported as mean ± SD (minimum, maximum) (or median [IQR], for non-normally distributed variables). *p*-values of paired *t*-tests (or Wilcoxon signed-rank tests, for non-normally distributed paired differences) between experts’ annotations and algorithm’s predictions are reported.

Classification Strategy	Time in Sedentary (h/day), *p*	Time in Light (h/day), *p*	Time in MVPA (h/day), *p*	Time in Non-Sedentary (h/day), *p*
Experts’ annotations	6.60 ± 2.56 (0.042, 13.6)	2.74 [1.68, 4.36]	0.504 [0.174, 1.12]	3.46 [2.25, 5.06]
Algorithm #1	7.20 ± 2.56 (0.042, 14.5), *p* < 0.001	1.69 [1.11, 2.72], *p* < 0.001	1.12 [0.701, 1.80], *p* < 0.001	3.01 [1.93, 4.41], *p* < 0.001
Algorithm #2	7.45 ± 2.52 (0.038, 14.59), *p* < 0.001 *	8.90 [7.32, 10.5], *p* < 0.001 *	1.58 [1.06, 2.26], *p* < 0.001	2.80 [1.86, 3.82], *p* < 0.001 *
Algorithm #3	5.94 [3.49, 7.91], *p* = 0.025	2.78 [1.85, 3.67], *p* = 0.036	0.979 [0.401, 2.62] *p* < 0.001	4.20 [2.65, 6.28], *p* = 0.025

Algorithm #1: Adamowicz et al.; Algorithm #2: Staudenmayer et al.; Algorithm #3: Montoye et al.; MVPA: moderate-to-vigorous physical activity; IQR: Interquartile Range; * indicates normally distributed paired differences.

**Table 2:** Daily time spent in different physical activity categories (h/day) across classification strategies, reported as mean ± SD (minimum, maximum) across participants. *p*-values of paired *t*-tests between experts’ annotations and algorithm’s predictions are reported.

Binary Classifier	Algorithm	Accuracy	Sensitivity	Specificity	Precision	F1-Score	Weighted Accuracy
Sedentary vs. Non-Sedentary	Algorithm #1	0.809 ± 0.080	0.888 ± 0.097	0.651 ± 0.144	0.804 ± 0.125	0.838 ± 0.096	0.770 ± 0.077
	Algorithm #2	0.798 ± 0.077	0.901 ± 0.079	0.604 ± 0.147	0.787 ± 0.130	0.833 ± 0.093	0.752 ± 0.073
	Algorithm #3	0.651 ± 0.212	0.666 ± 0.284	0.621 ± 0.220	0.725 ± 0.213	0.669 ± 0.239	0.643 ± 0.192
Light vs. Non-Light	Algorithm #1	0.741 ± 0.101	0.367 ± 0.092	0.886 ± 0.077	0.582 ± 0.179	0.438 ± 0.107	0.627 ± 0.053
	Algorithm #2	0.300 ± 0.113	0.731 ± 0.132	0.123 ± 0.096	0.260 ± 0.141	0.363 ± 0.148	0.427 ± 0.080
	Algorithm #3	0.717 ± 0.131	0.482 ± 0.190	0.808 ± 0.136	0.521 ± 0.254	0.478 ± 0.208	0.645 ± 0.128
MVPA vs. Non-MVPA	Algorithm #1	0.895 ± 0.074	0.595 ± 0.279	0.916 ± 0.070	0.355 ± 0.293	0.453 ± 0.256	0.755 ± 0.148
	Algorithm #2	0.855 ± 0.078	0.608 ± 0.281	0.872 ± 0.078	0.274 ± 0.243	0.385 ± 0.237	0.740 ± 0.146
	Algorithm #3	0.804 ± 0.174	0.294 ± 0.271	0.846 ± 0.173	0.195 ± 0.250	0.232 ± 0.222	0.574 ± 0.151

Algorithm #1: Adamowicz et al.; Algorithm #2: Staudenmayer et al.; Algorithm #3: Montoye et al.

## Data Availability

The dataset was collected by the University of Oxford, publicly available at: https://ora.ox.ac.uk/objects/uuid:99d7c092-d865-4a19-b096-cc16440cd001 (accessed on 2 April 2025). The dataset was used in accordance with its licensing terms. The original dataset creators bear no responsibility for the analyses or interpretations presented here, and their inclusion does not imply endorsement of this work.

## Abbreviations

The following abbreviations are used in this manuscript:

6MWT	Six-Minute Walk Test
AUROC	Area Under the Receiver Operating Characteristic Curve
CI	Confidence Interval
DHTs	Digital Health Technologies
EMA	European Medicine Agency
ENMO	Euclidean Norm Minus One
FDA	U.S. Food and Drug Administration
FDR	False Discovery Rate
FN	False Negatives
FP	False Positives
IQR	Interquartile Range
LoA	Limits of Agreement
MET	Metabolic Equivalent of Task
MVPA	Moderate-to-Vigorous Physical Activity
R	Pearson’s Correlation
RF	Random Forest
SD	Standard Deviation
SKDH	SciKit Digital Health
SPPB	Short Physical Performance Battery
SVM	Support Vector Machine
TN	True Negatives
TP	True Positives
TUG	Timed-Up-And-Go
WHO	World Health Organization
